# A red tide in the pack ice of the Arctic Ocean

**DOI:** 10.1038/s41598-019-45935-0

**Published:** 2019-07-02

**Authors:** Lasse M. Olsen, Pedro Duarte, Cecilia Peralta-Ferriz, Hanna M. Kauko, Malin Johansson, Ilka Peeken, Magdalena Różańska-Pluta, Agnieszka Tatarek, Jozef Wiktor, Mar Fernández-Méndez, Penelope M. Wagner, Alexey K. Pavlov, Haakon Hop, Philipp Assmy

**Affiliations:** 1 Norwegian Polar Institute, Fram Centre, Tromsø, Norway; 2 Department of Biological Sciences, University of Bergen, Bergen, Norway; 3 Polar Science Center, Applied Physics Laboratory, University of Washington, Seattle, WA USA; 4 Department of Physics and Technology, University of Tromsø - The Arctic University of Norway, Tromsø, Norway; 5 Alfred Wegener Institute Helmholtz Center for Polar and Marine Research, Bremerhaven, Germany; 6 Institute of Oceanology, Polish Academy of Sciences, Sopot, Poland; 7 Biological Oceanography, GEOMAR Helmholtz Centre of Ocean Research Kiel, Kiel, Germany; 8 Norwegian Ice Service, Norwegian Meteorological Institute, Tromsø, Norway; 9 Akvaplan-niva, Fram Centre, Tromsø, Norway; 10 Department of Arctic and Marine Biology, Faculty of Biosciences, Fisheries and Economics, University of Tromsø - The Arctic University of Norway, Tromsø, Norway

**Keywords:** Ecosystem ecology, Environmental health

## Abstract

In the Arctic Ocean ice algae constitute a key ecosystem component and the ice algal spring bloom a critical event in the annual production cycle. The bulk of ice algal biomass is usually found in the bottom few cm of the sea ice and dominated by pennate diatoms attached to the ice matrix. Here we report a red tide of the phototrophic ciliate *Mesodinium rubrum* located at the ice-water interface of newly formed pack ice of the high Arctic in early spring. These planktonic ciliates are not able to attach to the ice. Based on observations and theory of fluid dynamics, we propose that convection caused by brine rejection in growing sea ice enabled *M. rubrum* to bloom at the ice-water interface despite the relative flow between water and ice. We argue that red tides of *M. rubrum* are more likely to occur under the thinning Arctic sea ice regime.

## Introduction

In the high Arctic the relative contribution of ice algae to total primary production can be up to 60% because the snow covered perennial pack ice cover efficiently shades the under-ice water column, thus limiting phytoplankton growth^1–3^. During the ice algal spring bloom the highest biomass is usually found at the bottom 2–3 cm of sea ice in the interstitial environment of the skeletal layer forming as the ice grows. Here the cumulative light energy input is relatively high and nutrients are supplied from the underlying water^4–6^. Previously proposed physical mechanisms for colonization of the skeletal layer are harvesting or scavenging by frazil ice crystals, waves that push algae into the ice^7^, and the skeletal layer acting as a comb sieving algae from the water^5^.

Between the ice and the water column there is almost always a relative motion, due to water currents, and in the case of pack ice, wind-driven movement of the ice. Momentum transfer creates a boundary layer with decreasing velocity but increasing shear towards the ice under-surface, with laminar flow closest to the ice and turbulent flow outside^8^. From the benthic environment it is known that shear inhibits the colonization of surfaces by algae^9,10^. Typically the algae colonizing benthic surfaces can attach to the substrate and pennate diatoms are often dominating^11^. The same is true for sea ice^12,13^. Pennate diatoms excrete mucilage that enables them to adhere to and, in the case of raphe-bearing pennate diatoms, to move on the ice surface^14–16^. Motile algae without the capacity to adhere to the sea ice matrix are prone to displacement, presumably limiting biomass accumulation at the ice-water interface.

During the Norwegian young sea ice drift study (N-ICE2015) north of Svalbard^17^, we observed a dense bloom of the phototrophic ciliate *Mesodinium rubrum* (aka *Myrionecta rubra*) located at the ice-water interface of growing young ice (YI), in a lead that opened and refroze in late April and early May 2015. The bloom of *M. rubrum* at the ice-water interface can be likened to a red tide, well known for this species at lower latitudes^18^. To our knowledge this is the first observation of an ice-associated red tide of *M. rubrum* in the Arctic Ocean. *M. rubrum* is a motile planktonic species that, unlike e.g. diatoms and surface associated ciliates, cannot attach to the ice matrix^19,20^. Then, how can these ciliates remain stationary at the ice-water interface despite the flow? We calculated the theoretical thickness of the laminar boundary layer with the observed relative velocity and concluded that it is unlikely that the ciliates could reside within it. However, the red tide of *M. rubrum* coincided exactly with the period of ice growth, and we propose that in growing YI convection resulting from brine rejection compensated by seawater inflow interrupted the laminar boundary layer and allowed *M. rubrum* to stay in the ice-water interface as long as the ice was growing. The proposed hydrodynamic model provides a mechanistic explanation for the occurrence of blooms of motile algae below drifting pack ice.

## Results

### Study area, sea ice and water column conditions

**Figure 1:**
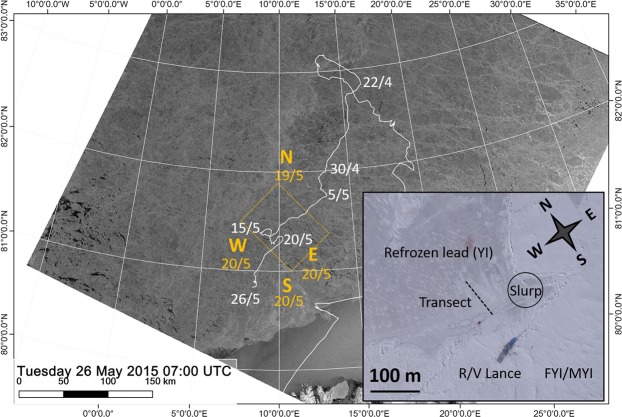
RADARSAT-2 image from 26 May 2015 showing the sea ice distribution in the study area north of Svalbard with the drift track of Floe 3 (white line) superimposed. The regional survey by helicopter 60 km north and 50 km east, south and west of R/V *Lance* on 19 and 20 May, respectively, are shown in yellow. The yellow rectangle indicates the area covered by an ALOS-2 radar scene that was used to classify ice types in a wider area around R/V *Lance* on 18 May (see Supplementary section 2). Inset is an aerial photo of the study site on Floe 3, showing a part of the refrozen lead and the area where divers took slurp samples from the ice-water interface, and the approximate position of the ice coring transects. RADARSAT-2 image provided by NSC/KSAT under the Norwegian-Canadian RADARSAT agreement. RADARSAT-2 Data and Products © Maxar Technologies Ltd (2015). All Rights Reserved. RADARSAT is an official mark of the Canadian Space Agency. The inset aerial image was taken on 23 May 2015 by V. Kustov and S. Semenov of the Arctic and Antarctic Research Institute, St. Petersburg, Russia.

The observations reported here are from the N-ICE2015 expedition lasting from January to June 2015, when R/V *Lance* drifted repeatedly with pack ice floes from about 83°N northwest of Svalbard south or southwestwards towards the ice edge at around 81°N^17^. We refer to the four ice drifts as Floes 1 to 4. This study is mainly from the drift of Floe 3 from 20 April to 6 June (Fig. 1), with some additional observations from Floe 4, which was followed from 8 to 22 June and started closer to the ice edge^17,21^, and measurements of the vertical flux of algae from all four floes. On Floe 3 we sampled young ice (YI) in a refrozen lead along a transect of five ice stations (Fig. 1). The lead was approximately 400 m wide and opened up on 23 April, started to freeze on 26 April and was completely ice covered by 1 May. At the first sampling on 6 May, the ice was 15 cm thick and it grew to a maximum of 27 cm around 18 May. Subsequently the ice melted to a thickness of 20 cm before it broke up on 3 June. The average snow depth on the refrozen lead during the whole period was 3.5 cm^22^. In addition, we took samples from thicker surrounding ice, which was classified to be either first year ice (FYI) or multiyear ice (MYI). The thick ice had a modal ice thickness of 1.46 ± 0.66 m and snow thickness of 0.39 ± 0.21 m according to a local area survey^23^. The loss in total thickness of snow and ice in the period from 27 April to 4 June was 6 and 3 cm per 30 days for MYI and FYI, respectively^23^, indicating minor changes in the ice pack surrounding the refrozen lead. Under the FYI and MYI the irradiance was 1–10 µmol photons m^−2^ s^−1^, and under YI on average 114 µmol photons m^−2^ s^−1^ ^21,22^. The mixed layer depth of the water column below the ice was approximately 50 m throughout the study period^24^. During this study nutrients (phosphate, nitrate and silicate) were always available in excess in the under-ice water^21^.

### *Mesodinium rubrum* across sea ice habitats and water column

**Figure 2:**
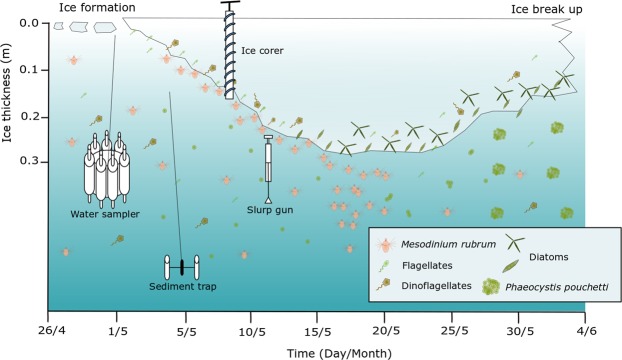
Timeline of the observations of *M. rubrum* in YI, at the ice-water interface and the underlying water column, with the sampling methods indicated. Divers took samples from the ice-water interface with the slurp gun. Sea ice diatoms became dominating in the ice algal community after 20 May. In addition to *M. rubrum* various flagellates were present in the water column. See Kauko *et al*.^13^ for a detailed description of the ice-algal succession and Assmy *et al*.^25^ for a description of an under-ice bloom of *Phaeocystis pouchetii* in the water column starting around 25 May.

**Figure 3:**
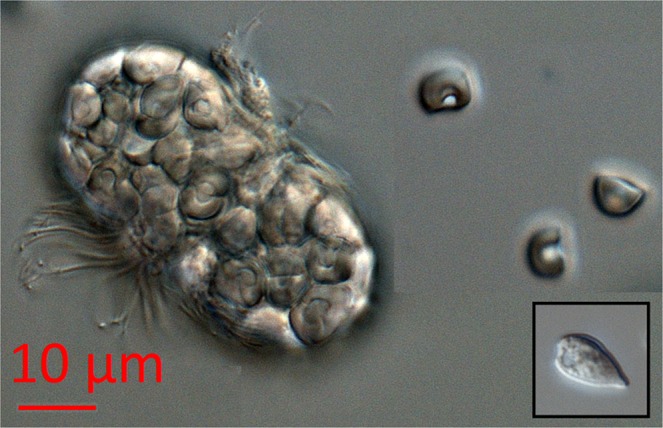
*Mesodinium rubrum* cell and three free chloroplasts originating from *M. rubrum* cells at the right side. Note same type of cells inside the *M. rubrum* cell. Inset is an image of an unidentified cryptophyte from the same sample. *M. rubrum* ingests cryptophyte algae, sequester their chloroplasts and subsequently use them for photosynthesis^49^. All images were taken at 200x magnification.

**Figure 4:**
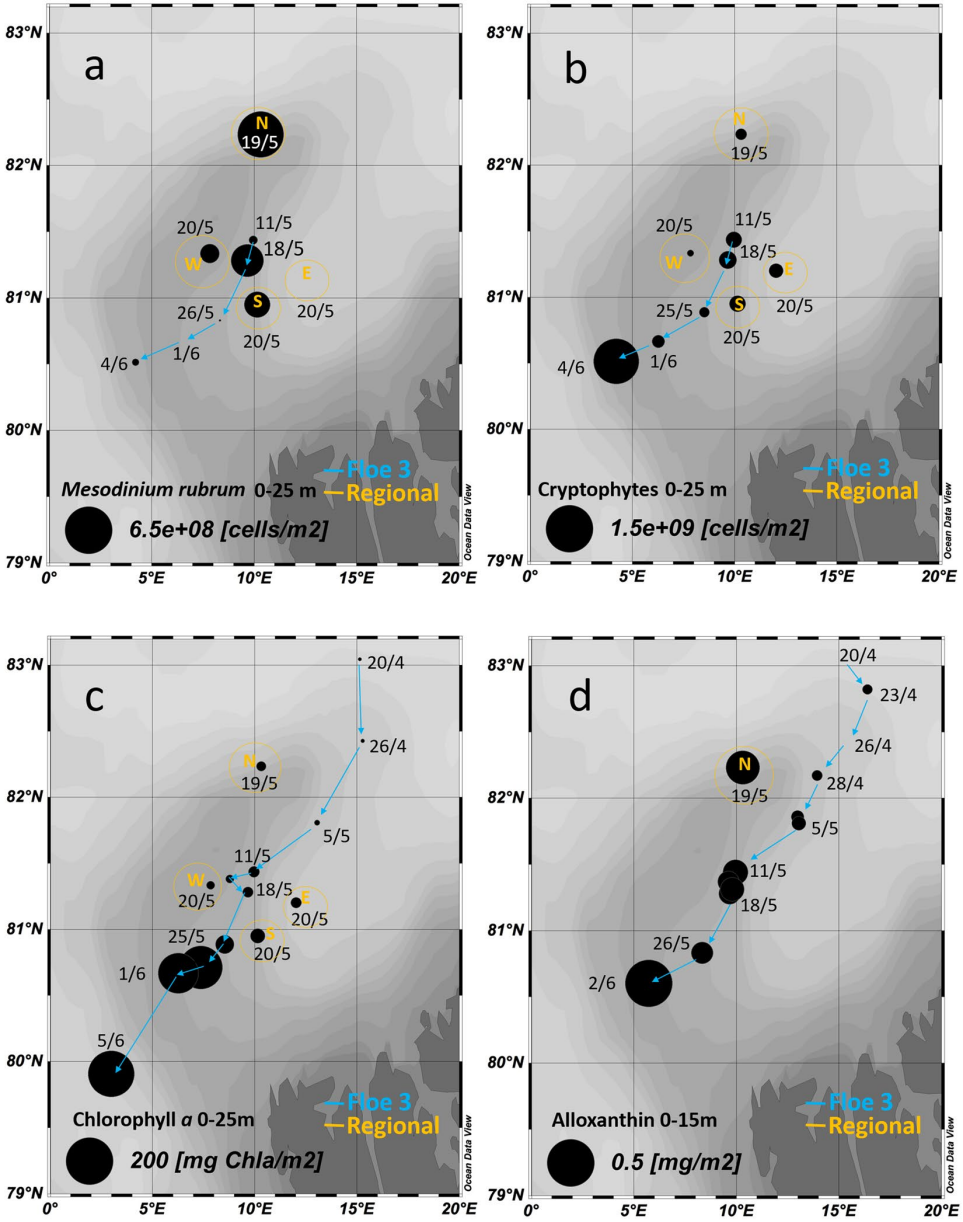
Temporal and spatial map of water column abundance of *Mesodinium rubrum* (**a**) and cryptophytes (cells m^−2^) (**b**), integrated (0 to 25 m) chlorophyll *a* (Chl *a*) (mg m^−2^) (**c**) and integrated (0–15 m) alloxanthin (mg m^−2^) (**d**) standing stocks during the drift of Floe 3 (blue). In yellow circles the values for the regional sampling north, east, south and west of R/V *Lance* on 19 and 20 May.

*Mesodinium rubrum* was detected in young ice (YI) of the refrozen lead throughout the study period with abundance in the range of 0.3 to 15.7 × 10^6^ cells m^−2^ (Table 1). The highest abundance (2.9 to 211 × 10^6^ cells m^−2^) was observed in slurp gun samples taken by divers from the ice-water interface (Fig. 2). The bloom was visible as a faint coloring of the ice undersurface, and the sampling with the slurp gun indicated that the algal layer had a thickness of <1 mm and was stationary at the interface. Because the area sampled with the slurp gun was known we could calculate cells per area. The per volume cell concentration in the slurp samples was in the range 2.3 × 10^4^ to 3.1 × 10^6^ cells L^−1^, but the slurp samples were diluted when surrounding seawater entered due to the suction. Free chloroplasts originating from burst *Mesodinium rubrum* cells (Fig. 3) were found in relatively high abundance (0.4 to 7.2 × 10^9^ chloroplasts m^−2^) in the bottom 10 cm of YI of the refrozen lead in the period 4 to 20 May, and then a rapid, two orders of magnitude decline after 20 May (Table 1). At the ice-water interface, the abundance of chloroplasts amounted to 9.2 × 10^7^ to 3.8 × 10^9^ m^−2^. Neither *M. rubrum* cells nor free chloroplasts were detected by microscopy in FYI or MYI (Table 1). In the water column the abundance of *M. rubrum* (Fig. 4a) peaked on 18 May with 3.1 × 10^8^ cells m^−2^, and a regional helicopter sampling revealed abundances between 1.0 and 6.0 × 10^8^ cells m^−2^ in a larger area around R/V *Lance* (Fig. 4a). The per volume concentration of *M. rubrum* in the water column was in the range of 2 to 52 × 10^3^ cells L^−1^. *M. rubrum* constituted up to 40% of the total cell abundance of the protist community (Fig. S1.3) on 18 May at R/V *Lance* and from the regional sampling on 19 and 20 May. *M. rubrum* cells were found in the sediment traps from 5, 25, 50 and 100 m depth on 26 April, 10 May, 18 May and at 5 m on 16 June (Table 2). The calculated vertical flux was in the order of 10^6^ cells m^−2^ d^−1^ at 5 to 50 m and 10^5^ cells m^−2^ d^−1^ at 100 m on 26 April, 10^6^ cells m^−2^ d^−1^ at all depths on 18 May and increased to 10^7^ cells m^−2^ d^−1^ at 5 and 25 m on 18 May. No *M. rubrum* was found in the traps on 29 May and 12 June, and a lower flux in the order 10^4^ cells m^−2^ d^−1^ was found at 5 m on Floe 4 on 16 June (Table 2). In the sediment traps deployed 1 m below the ice, a vertical flux in the order of 10^6^ cells m^−2^ d^−1^ was observed on 10 and 18 May, whereas no *M. rubrum* cells were found in the traps at this depth on 26 April, or after 18 May (Table 2). In Fig. 2 we show schematically our observations of *M. rubrum* in YI, at the YI ice-water interface, and in the water column.

**Table 1:** Temporal development of chlorophyll *a* (Chl *a*) and alloxanthin (Allo) standing stocks (mg m^−2^) in the ice-water interface of YI (average ± SE, n = 3 sites) and in ice cores of YI (average ± SE, n = 5 sites), FYI and MYI (n = 1) on Floe 3 (4 May–4 Jun 2015).

Date	Refrozen lead (YI) ice-water interface (slurp)					Refrozen lead (YI) ice cores^a^					FYI^b^		MYI^b^	
	Chl *a*	Allo	Crypto	*M. rub*	chloro	Chl *a*	Allo	Crypto	*M. rub*	chloro	Chl *a*	Allo	Chl *a*	Allo
4 May						1.0 ± 0.4	0.08 ± 0.05		14.2 ± 10.3	7164 ± 3515				
5						0.09 ± 0.03								
6	15.3	0.8				0.1 ± 0.02	0.01 ± 0.002		2.0 ± 0.09	823 ± 810				
7						0.3 ± 0.1	0.004 ± 0.001	2.3 ± 2.3	0.3 ± 0.3	635 ± 97				0.001
8	4.4													
10	9.4 ± 0.6	0.2 ± 0.1	0	2.9 ± 0.9^c^	92 ± 26^c^	0.7 ± 0.3	0.05 ± 0.02	0.4 ± 0.4	0.4 ± 0.4	2823 ± 1013				
12	4.5 ± 0.9		0	22 ± 5.8^c^	303 ± 85^c^	0.4 ± 0.06		2.5 ± 1.0		434 ± 334				
14	6.7 ± 1.1	1.5 ± 0.2	1.04	211 ± 40	3817 ± 725	0.3 ± 0.05		3.3 ± 0.3	0.6 ± 0.6		0.2	0.004	0.3	0.002
16						1.1 ± 0.1		0.5 ± 0.5	2.3 ± 1.2	2295 ± 1587				
18						1.2 ± 0.1	0.04 ± 0.01	4.5 ± 0.5						
20						2.9 ± 0.3	0.4 ± 0.2	8.5 ± 1.2	15.7 ± 3.3	3784 ± 1483				
21													0.6	0.008
22						1.4 ± 0.2	0.04 ± 0.003	2.9 ± 0.5	0.8 ± 0.5	485				
23											1.2	0.008		
24						1.6 ± 0.3	0.03 ± 0.009	6.9 ± 3.8		107 ± 67				
26							0.03 ± 0.01	8.8 ± 4.1	2.1 ± 1.1					
28												0.005	0.9	0.006
29						3.1 ± 0.3	0.03 ± 0.01			90 ± 34				
1 Jun						3.0 ± 0.6	0.05 ± 0.01							
3										67 ± 25				
4														0.003

From ice-water interface and ice cores of YI abundance (average 10^6^ cells m^−2^ ± SE, n = 3–5, 0 = below detecton limit) of cryptophytes (Crypto), *Mesodinium rubrum* (*M. rub*) and free *M. rubrum* chloroplasts (chloro) are shown. Cryptophytes are prey, supplying chloroplasts to *M. rubrum*^49^. Alloxanthin is a pigment produced by cryptophytes and is therefore also found in the chloroplasts of *M. rubrum*^26^.

^a^4 May–16 May: whole core, 20 May–3 Jun: bottom 10 cm.

^b^Bottom 10 cm of core.

^c^Errors denote confidence intervals for count precision according to Edler and Elbrächter^54^ used when n = 1.

**Table 2:** Vertical flux of *Mesodinium rubrum* (10^6^ cells m^−2^ d^−1^) at 5 depths (m). 30 Jan on Floe 1, 14 Mar on Floe 2, 26 Apr–29 May on Floe 3, and 12 and 16 Jun on Floe 4 of N-ICE2015 ice drifts.

Depth	30 Jan	14 Mar	26 Apr	10 May	18 May	29 May	12 Jun	16 Jun
1	0	0	0	3.78	5.05	0	n.d.	n.d.
5	0	0	5.37	2.41	28.9	0	0	0.05
25	0	0	6.49	6.93	12.5	0	0	0
50	0	0	4.51	6.55	7.37	0	0	0
100	0	0	0.18	3.99	5.23	0	0	0

0 = below detection limit, n.d. = no data. See Granskog *et al*.^17^ for drift tracks of all floes.

Abundances of cryptophyte algae, the prey and source of chloroplasts for *M. rubrum*, in the YI ranged from 0.4 to 8.8 × 10^6^ cells m^−2^ (Table 1). At the ice-water interface, cryptophytes were only detected once, on 14 May, and none were observed in FYI or MYI (Table 1). The abundance of cryptophytes in the water column was in the range of 0.3 to 2.1 × 10^8^ cells m^−2^ in May, but a higher abundance of 1.4 × 10^9^ cells m^−2^ was observed in June (Fig. 4b).

The highest chlorophyll a (Chl *a*) standing stock, a proxy for algal biomass, was measured at the ice-water interface of the refrozen lead with values in the range 0.9–15 mg m^−2^ for the period 6–14 May. The Chl *a* concentration in these slurp samples was in the range 7–117 mg m^−3^. In the same period, the Chl *a* standing stock in the ice cores was in the range of 0.09–1 mg m^−2^. The highest Chl *a* standing stock measured in ice was around 3 mg m^−2^ in the bottom 10 cm of the refrozen lead in late May and early June (Table 1). In the water column, the depth integrated (0–25 m) Chl *a* standing stock increased in early May from 1 mg m^−2^ to 9.7 mg m^−2^ on 18 May (Fig. 4c). The regional sampling by helicopter to the north (19 May), east, south and west (20 May) relative to R/V Lance revealed similar levels of 6–19 mg Chl *a* m^−2^ in a larger area at this time (Fig. 4c). The concentration of Chl *a* at this time never exceeded 0.5 mg m^−3^. From 25 May onwards, Chl *a* standing stocks increased by a factor of 10–20 (Fig. 4c), which was due to an under-ice bloom of *Phaeocystis pouchetii* described by Assmy *et al*.^25^. According to the ice classification performed on the ALOS-2 radar satellite scene YI made up 10.2% of the total area, thicker FYI or MYI constituted 84.4%, and open water 5.4% (Fig. S2.1). Upscaling to the 2800 km^2^ ALOS-2 scene area (Fig. 1) indicate that the Chl *a* in the thin (<1 mm) layer of the YI interface equals 4.3% of the total integrated amount found in the water column from 0 to 25 m depth.

Alloxanthin standing stocks, a proxy for cryptophyte and/or *Mesodinium rubrum* biomass^26^, were orders of magnitude higher at the ice-water interface of YI (0.2 to 1.5 mg m^−2^) than in the YI cores (0.004 and 0.08 mg m^−2^ with a peak of 0.4 mg m^−2^ on 20 May). In the FYI and MYI, the standing stocks of alloxanthin were even lower, in the range 0.001–0.008 mg m^−2^ (Table 1). The alloxanthin: Chl *a* ratio (mg: mg) was in the range of 0.01–0.03 in YI, except for ratios of 0.07–0.13 coinciding with Chl *a* peaks on 4, 10 and 20 May^13^, and up to 0.36 at the ice-water interface. In the water column alloxanthin standing stocks increased from winter levels of 0.02–0.04 mg m^−2^ to approximately 0.1 mg m^−2^ in the period 11–18 May (Fig. 4d). From the spatial sampling campaign on 19–20 May, we only have the northern point for alloxanthin, which showed 0.25 mg m^−2^, indicating that the increase was taking place over a larger area. The alloxanthin to Chl *a* ratio in the water column was between 0.02 and 0.06, and after 20 May <0.02.

In the slurp gun samples taken by divers at the ice-water interface *M. rubrum* and its chloroplasts dominated throughout the sampling period from 7–14 May, contributing 87–97% and 92–97% of the total protist abundance and carbon biomass, respectively (Fig. S1.2). In the YI cores the free *M. rubrum* chloroplasts totally dominated in abundance and constituted a large part of the carbon biomass until 20 May^13^. In addition flagellates constituted a significant fraction in early May while pennate diatoms gradually increased and became the dominating group in late May. See Kauko *et al*.^13^ for a detailed description of the succession of the protist community in the YI of the refrozen lead, and Olsen *et al*.^21^ for the surrounding FYI and MYI. The protist community in the water column during early May was dominated by dinoflagellates, flagellates and the “Other” group (Fig. S1.3) dominated by *Phaeocystis pouchetii*, ciliates other than *Mesodinium rubrum* and coccolithophorids (Supplementary Section 4). For most sediment trap samples, in which *M. rubrum* was found, the majority of the protist cells were *M. rubrum*, which constituted almost the entire carbon biomass (Fig S1.4). In Fig. 4 is shown a schematic summary of the observations in ice, ice-water interface and water column, with sampling methods indicated.

### Photosyntetic response to irradiance

The maximum quantum yield of fluorescence (Φ_PSIImax_) measured in slurp samples from the ice-water interface of the refrozen lead on 5–14 May was in the range 0.40–0.64 (Table 3). The range for the photosynthetic parameters derived from fitting the Webb equation to the rapid light curve measured were: photosynthetic efficiency (α) = 0.32–0.59 (µmol photons m^−2^ s^−1^)^−1^, maximum relative electron transfer rate (rETR_max_) = 72–198 (no unit), saturation irradiance (E_k_) = rETR_max_/α = 153–549 µmol photons m^−2^ s^−1^ (Table 3). The measured downwelling PAR irradiance at the ice-water interface of the refrozen lead reached a maximum of 114 µmol photons m^−2^ s^−1^ ^22^.

**Table 3:** Maximum quantum yield of fluorescence of photosystem II (Φ_PSIImax_) and the photosynthetic parameters from samples taken at the YI ice-water interface. rETR_max_: maximum relative electron transfer rate (no unit), α: photosynthetic efficiency (µmol photons m^−2^ s^−1^)^−1^, E_k_ = rETR_max_/α: photosynthetic saturation irradiance (µmol photons m^−2^ s^−1^). Average ± SE, n = 3.

Date	Φ_PSIImax_	rETR_max_	α	E_k_
5 May	0.64 ± 0.11	128 ± 12	0.59 ± 0.03	219 ± 22
6	0.57 ± 0.01	118 ± 24	0.50 ± 0.02	239 ± 55
10	0.40 ± 0.05	72 ± 20	0.46 ± 0.07	153 ± 20
12	0.54 ± 0.03	198 ± 30	0.51 ± 0.05	383 ± 35
14	0.46 ± 0.07	168 ± 46	0.32 ± 0.10	549 ± 33

### Ice-ocean boundary layer dynamics

**Figure 5:**
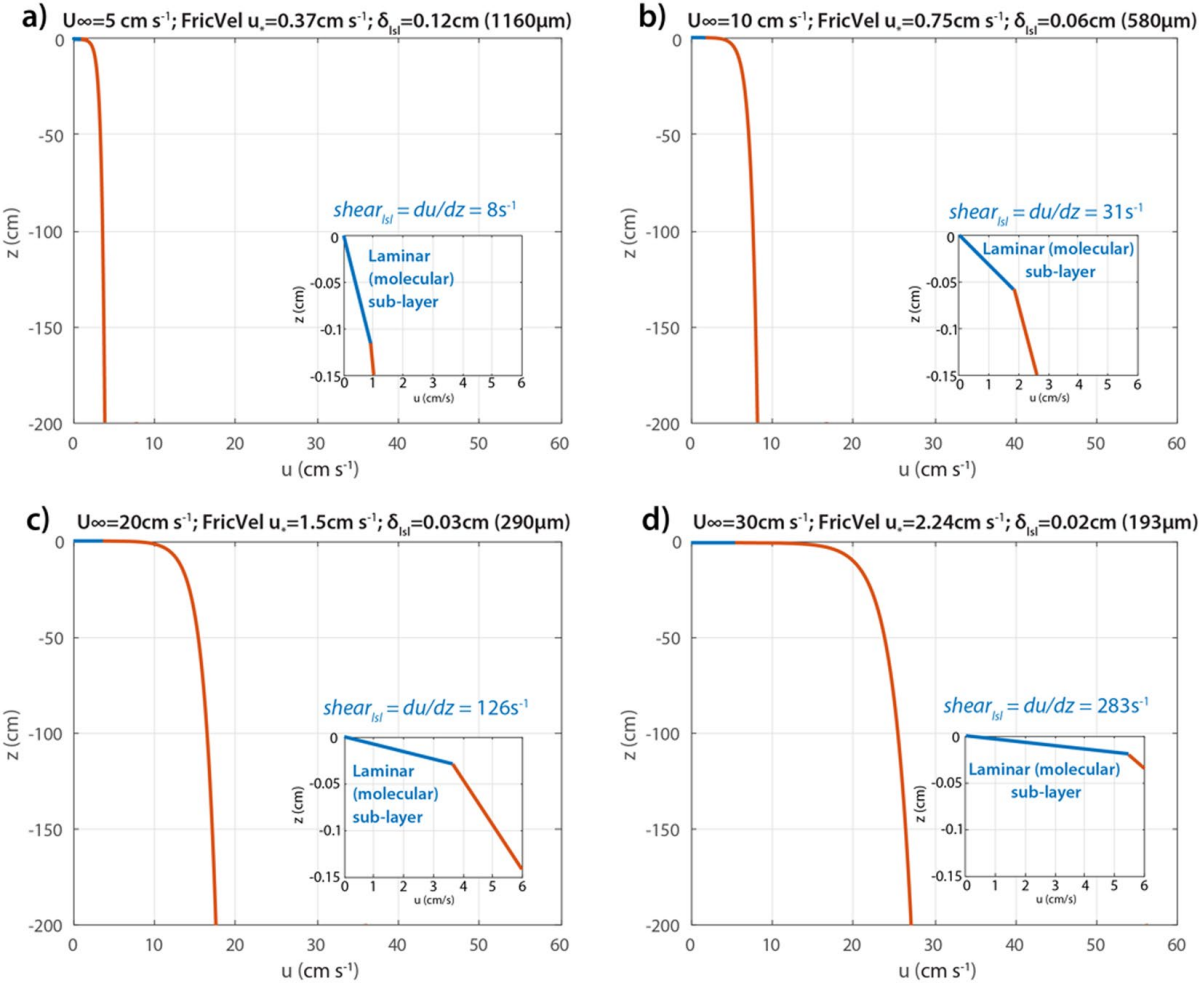
Velocity profiles in the laminar sub-layer (blue) and the logarithmic layer (red) below assumed smooth ice, considering free stream velocities of (**a**) U_*∞*_ = 5 cm s^−1^; (**b**) U_*∞*_ = 10 cm s^−1^; (**c**) U_*∞*_ = 20 cm s^−1^; and (**d**) U_*∞*_ = 30 cm s^−1^. Inserts highlight the laminar sub-layer region.

The average relative velocity between ice and water was 11 cm s^−1^ (Fig. S1.1). Figure 5 shows the velocity profiles from the sea ice boundary down to 2 m depth, including both the laminar sub-layer and the turbulent logarithmic layer. A zoom of the upper 0.15 cm right below the sea ice gives details of the laminar sub-layer velocity structure and thickness. For a free-stream velocity of U_*∞*_ = 10 cm s^−1^ (Fig. 5b), the thickness of the laminar sub-layer is *δ*_*lsl*_ = 0.06 cm. During times with free-stream velocities lower than average (Fig. 5a), the thickness of the laminar sub-layer is larger (*δ*_*lsl*_ = 0.12 cm for U_*∞*_ = 5 cm s^−1^), whereas during events of stronger free-stream velocities (Fig. 5c,d), the thickness of the laminar sub-layer is much smaller (*δ*_*lsl*_ = 0.03 cm and *δ*_*lsl*_ = 0.02 cm for U_*∞*_ = 20 cm s^−1^ and U_*∞*_ = 30 cm s^−1^, respectively). In Supplementary Material Section 4 we describe how under-surface roughness can affect the boundary layer dynamics.

## Discussion

The 7–117 mg Chl *a* m^−3^ we measured in the slurp samples from the YI interface layer is 14–234 times higher than the concentration in the water column (<0.5 mg Chl *a* m^−3^), which was at the level of non-bloom concentrations reported from the North Atlantic^27^. The interface bloom can be likened to the red tides of *M. rubrum* often observed at lower latitudes, where the Chl *a* concentration can be >100 mg m^−3^ and abundance up to 10^6^ cells L^−1^ ^28^. To our knowledge, ours is the first observation of a pack ice associated red water bloom. The only published observation of a red tide in the Arctic Ocean is from ice-free, coastal waters near Barrow, Alaska in September 1968, caused by an unidentified ciliate similar to, but not identical with *M. rubrum*^29^. The abundance in the bottom 10 cm of YI (0.3 to 15.7 × 10^6^ cells m^−2^) is comparable to some other observations. Up to 1.6 × 10^6^ cells m^−2^ of *M. rubrum* was observed in the bottom 2–4 cm of 30–40 cm thick FYI in the Saroma-ko lagoon in Hokkaido, with an integrated abundance in the water column under the ice from 0 to 1 m depth of 3.4 × 10^6^ cells m^−2^ ^30^. Likewise, when *M. rubrum* was observed at abundance 2 × 10^5^ cells m^−2^ in the bottom 2–4 cm of 1.5–2 m thick FYI in the Canadian Arctic, the average abundance in the water column below the ice down to 8 m was 0.14 × 10^3^ cells L^−1^ ^31^. The maximum abundance we measured in the YI ice-water interface (211 × 10^6^ cells m^−2^) was considerably higher than these observations.

*M. rubrum* cells are known to be fragile and difficult to preserve. The mix of glutaraldehyde and formaldehyde used during the N-ICE campaign was chosen in order to preserve the highest possible fraction of the entire protist community, but might not be the best method to preserve *M. rubrum*^18^. The high abundance of chloroplasts originating in *M. rubrum* in the ice core samples (Table 1), indicate that cells had disintegrated. The melting of the ice cores could also have caused ciliates to burst^32^ Use of the free chloroplasts as a tracer of *M. rubrum* relies on the accurate identification of them. The morphology of the free chloroplast was very similar to those we observed inside of *M. rubrum* cells (Fig. 3), and agrees well with previous descriptions^33^.

According to the quantum yield of fluorescence (0.40 to 0.64) the protists, mainly *M. rubrum*, were in a good physiological condition^34^, with active photosynthesis at the YI ice-water interface. Under the FYI and MYI with 20–50 cm of snow the irradiance was only 1–10 µmol photons m^−2^ s^−1^ ^21^. This ice type covered most of our study area (Fig. S2.1). The *M. rubrum* bloom was confined to the ice-water interface of the YI in the refrozen lead, where the irradiance was higher, on average 114 µmol photons m^−2^ s^−1^ ^22^. Moeller *et al*.^35^ showed that *M. rubrum* acclimates to the irradiance level so that the saturation irradiance for photosynthesis (E_k_) is similar to the irradiance they grow under. We measured E_k_ > 153 µmol photons m^−2^ s^−1^, indicating they were growing stationary at the YI ice-water interface. This E_k_ is similar to what Stoecker *et al*.^36^ found for *M. rubrum* in temperate waters, and what McMinn and Hegseth^37^ found for surface phytoplankton in the Arctic Ocean north of Svalbard in spring.

Under YI is a near optimal place with regards to light, but can *M. rubrum* cells actively position themselves under the thin ice, or is there some external physical mechanism keeping them there? The swimming speed of *M. rubrum* is approximately 0.16 mm s^−1^ ^19^, whereas the average relative velocity of ice vs. water was 0.11 m s^−1^ (Fig. S1.1), so they could certainly not outswim the ice. Previously proposed mechanisms like scavenging of cells by frazil ice in the water column and waves pushing the cells into the ice^7^ seems unlikely here because both processes are most active when there is open water or still unconsolidated ice, whereas our bloom took place at the ice-water interface of consolidated YI. Sieving of the water column by the protruding ice crystals of the skeletal layer^5^ seems more likely to work for sticky algae like diatoms^38^ than for the fast swimming/jumping^19,39^ and, to our knowledge, non-sticky *M. rubrum*.

**Figure 6:**
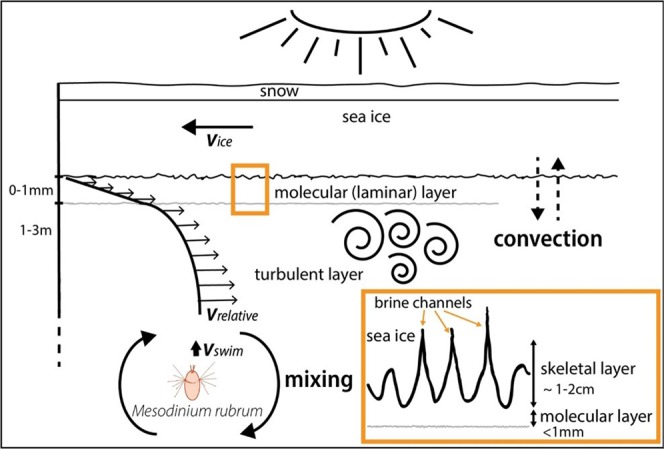
The various forces acting upon a cell of *M. rubrum*, to modify its position relative to the drifting sea ice. The relative motion between ice and water (V_relative_) creates a boundary layer where the laminar part has a velocity increasing linearly from zero at the surface while the turbulent part exhibits a logarithmic increase in relative velocity. *M. rubrum* is phototactic, swimming upwards to higher irradiance. (V_swim_) Water column mixing can assist or counteract the upward movement. The skeletal layer formed at the bottom of growing sea ice consists of ice crystal lamellae interspersed by brine channels and tubes. Brine rejection compensated by inflowing seawater creates convection that may contribute to keep *M. rubrum* cells there. See discussion and Supplementary section 3 for details.

Is it possible that in the boundary layer close to the ice undersurface the relative motion of water and ice is so slow that *M. rubrum* cells can remain stationary there? Boundary layer shear is known to affect algal colonization of the benthic environment^40^, and although the boundary layer under sea ice is well studied in other physical contexts^8,41^, there seems to be no studies on how it affects algal colonization. Figure 6 shows a schematic compilation of the various forces acting on a cell of *M. rubrum* to modify its position relative to the ice. In a simplified scenario of a smooth sea ice bottom and no sea-ice melt or growth, and with the range of relative velocities between ice and water that we observed (Fig. S1.1), the thickness of the laminar part of the boundary layer would theoretically be 0.2–1.2 mm (Fig. 5). *M. rubrum* cells have a maximum width of 20 µm and length of 40 µm^27^, and move in jumps of 0.16 mm. A typical jumping rate is 1 s^−1^, and thus, the effective swimming velocity is 0.16 mm s^−1^ ^19^. This implies that the laminar layer was 3–4 jumps thick at the average ice-water relative velocity. The shear in the layer is in the range 8–283 s^−1^, from lowest to highest free stream velocity (Fig. 5). In addition to being phototactic, *M. rubrum* is also rheotactic, and a shear of 1–3 s^−1^ is enough to trigger an escape response according to Fenchel and Hansen^19^. In addition to the thinness of the layer and the high shear, the velocity reached within the laminar layer was 1 cm s^−1^ or higher (Fig. 5), i.e. above the swimming speed of *M. rubrum*. Thus, it seems unlikely that a bloom at the ice-water interface can be maintained within the laminar boundary layer.

Macroscopically the cores appeared relatively smooth at the bottom, with roughness on the mm scale. If the roughness created hydrodynamically rough conditions, i.e. allowed turbulence to reach the ice surface, it is unlikely that it helped *M. rubrum* cells to stay in the ice-water interface because the laminar layer would become even thinner or disappear completely (Supplementary Section 3). Roughness did not help sticky diatoms to colonize benthic surfaces^9^, then it might be even more unlikely to help non-sticky ciliates.

The bloom at the YI interface disappeared abruptly over a few days after 20 May (Fig. 2). At this time there was still a surplus of inorganic nutrients in the water below the ice, and ice diatoms continued to grow at the interface for two weeks until the floe broke up^13,21^, i.e. nutrient limitation was not causing the disappearance. *M. rubrum* was at saturating abundance for copepods in the interface if they were able to exploit this food source, but we observed no response in grazer abundance or aggregation of grazers at the interface. Thus, it is unlikely that grazing terminated the bloom.

Noteworthy, over the entire period we observed the interface bloom the YI was growing. The disappearance of the bloom coincided with the cessation of ice growth (Fig. 4), suggesting that ice growth might create physical conditions favorable to keep *M. rubrum* at the interface. Almost no ice growth was observed in FYI and MYI in early May^42^ due to the insulating effect of the thick snow cover^43^, and *M. rubrum* was not found there (Table 1). During ice growth a porous skeletal layer is formed at the bottom of the ice with pockets and tubes, which can be 1–3 cm long and with a diameter up to 0.5 mm^44^. In this process brine is rejected by gravity drainage^45^. The decrease in bulk ice salinity observed indicates that this happened as predicted when the refrozen lead ice formed^42^. Brine drainage from the ice is compensated by an inflow of seawater, forming convection cells^46–48^. It is possible that this skeletal layer convection disrupts the laminar boundary layer (J. Morison personal communication) and helps *M. rubrum* cells to remain in the skeletal layer, maybe assisted by their own upwards, phototactic swimming (Fig. 6). In addition convection renews the water in the skeletal layer, supplying nutrients from the water column^4^. This might also supply cryptophyte prey and thus new chloroplasts to *M. rubrum*^49^.

When the refrozen lead ice stopped growing around 20 May (Fig. 2), it follows that brine drainage, and thus also convection stopped^45^. At this point the physical factors at the ice-water interface were presumably dominated by the boundary layer dynamics, which, as discussed above, did not help *M. rubrum* to stay in the interface. In contrast, the interstitial ice diatom community continued to grow and reached maximal biomass in late May after ice melt had started^13,21^, illustrating the benefit of being adhered to the ice^14,15,50^.

The highest abundance of *M. rubrum* in the water column was observed on 18 May (Fig. 4a) coinciding with its highest vertical flux (Table 2). This could be related to a mass release of *M. rubrum* from the ice due to the cessation of ice growth, as discussed above. The tendency of *M. rubrum* to migrate and aggregate in the water column makes it difficult to get an accurate measure of the abundance with the fixed sampling depths during the CTD casts^18^. The sediment traps capture cells during 1–2 days and therefore might be a more reliable device for detecting *M. rubrum*. According to the vertical flux (Table 2) about 10% of the standing stock from 0–25 m was captured per day on 18 May, and the apparent sinking velocity was 0.28 m d^−1^ at 25 m. The sinking speed of a resting *M. rubrum* cell is 0.7 m d^−1^ according to Fenchel and Hansen^19^. It is reasonable that these motile, phototactic ciliates had a low sinking velocity, whereas the migratory behavior might lead *M. rubrum* cells to swim into the traps^51^.

The regional sampling showed similar abundances in a larger area surrounding R/V *Lance* on 19 and 20 May (Fig. 4a), suggesting that the *M. rubrum* red tide was not restricted to the refrozen lead we studied but was a regional phenomenon. The drift track of many ice-tethered buoys in the area around R/V *Lance* for the same time period indicated that the wind speed and direction was the same in the entire area covered by the ALOS-2 scene^52^. Thus, it is reasonable to assume that the temperature conditions were similar, and therefore that all YI was growing at this time, facilitating a large scale ice-water interface red tide of *M. rubrum* in an area exceeding 2800 km^2^ (Fig. 1). According to the ice type classification in the ALOS-2 satellite radar scene from 18 May (Fig. S2.1), which partly covered the regional sampling campaign by helicopter (Fig. 1), YI made up 10.2% of the total area. The Chl *a* in the YI ice-water interface equaled 4.3% of the total amount found in the water column from 0 to 25 m depth, which is considerable considering the huge volume of water and the thinness of this layer (<1 mm).

The ongoing regime shift towards a thinner, more dynamic ice cover in the Arctic Ocean, with more lead formation^25^ can promote ephemeral blooms of *M. rubrum* below growing young ice. It is important to improve our understanding of the mechanisms enabling ice-associated blooms of different algal taxa. Shifts in species composition at the base of the ice-associated ecosystem is an indicator of change, and are likely to have cascading effects on the Arctic marine food web and the biological carbon pump of the Arctic Ocean.

## Methods

### Current measurement

A medium-range vessel-mounted broadband 150 kHz acoustic Doppler current profiler (ADCP) from Teledyne RD Instruments was used to measure current speed and direction below the ice. The profiles were hourly-averaged in 8-m vertical bins and the first bin was centered at 23 m^24^. The current speed and direction at 23 m depth were used to calculate the current relative to the ice floe based on ship navigation data. The relative current speed measured between the water column and the sea ice was in the range 0–0.3 m s^−1^, with an average of 0.11 m s^−1^ (Fig. S1.1). The apparent northward direction of the water was mainly due to the faster southward movement of the ice driven by prevailing northerly winds^52^. To study the variability of water column properties in a larger area around R/V *Lance*, samples were taken at the end of helicopter transects about 60 km north of the ship on 19 May, and about 50 km east, west, and south of the ship on 20 May (Fig. 3).

### Sample collection and analysis

Seawater samples for chlorophyll *a* (Chl *a*), pigment composition and protist counts were collected with a rosette water sampler with 8 L Niskin bottles deployed from the ship or with 3.5 L Niskin bottles on a rosette deployed from the ice. Samples were taken at 5, 25, 50 and 100 m depths from the ship, and from 2, 5 and 15 m with the on-ice system. During a regional sampling campaign by helicopter on 19 and 20 May water samples were taken manually with a Limnos water collection bottle closed with a messenger (Limnos. pl) at 5, 15 and 25 m depth. To obtain depth integrated values of Chl *a* or abundance we used the trapezoid method. Because we had no measurements from 0 m we set the values to equal those at 5 m depth.

Three ice-tethered sediment traps (KC Denmark) were deployed on a rope stretched under the YI horizontally at 1 m depth. In addition, four sediment traps were deployed vertically at 5, 25, 50, and 100 m depth, respectively, along a mooring attached to the ice. The deployment time was between 36 and 72 h, usually 48 h. To avoid loosing sample water from the traps during deployment and recovery, the trap cylinders were filled with filtered seawater, made hypersaline (i.e. more dense) by adding sodium chloride, before deployment. Each trap had two cylinders with internal diameter 7.2 cm and height 45 cm, with no baffle at the top. At sampling the water from both cylinders were combined into one sample. Copepods and other zooplankton were removed before taking samples for algal taxonomy. Sinking flux for protists was calculated from cell concentration in the traps, trap volume and area, and trap deployment time.

Samples from the sea ice were taken with 9 and 14 cm diameter ice corers (Mark II coring system, KOVACS enterprise, Roseburg, USA). The cores were cut into 10 or 20 cm sections, put in cleaned opaque plastic containers and melted during 18–24 hours at room temperature without seawater buffer on board the ship, according to Rintala *et al*.^31^.

Samples from the ice-water interface under the refrozen lead were taken by scuba divers using a modified 3.5 L Trident® suction gun (slurp gun). The front nozzle was oblique so that it was possible to fill the gun while moving it along the undersurface of the sea ice. The surface area sampled was 5 × 54 cm for a full slurp gun and this area was used to transform cells per volume in the sample to cells per area.

For protist taxonomy analysis and cell counts, 190 mL from Niskin bottles, slurp gun, sediment traps, or melted ice cores were transferred into 200 mL brown glass bottles and fixed with an aldehyde mixture consisting of glutaraldehyde at a final concentration of 0.1% and hexamethylenetetramine-buffered formaldehyde at a final concentration of 1% (vol:vol). The samples were stored dark and cool until analysis. Protists were counted with an inverted Nikon Ti-U light microscope (Nikon TE300 and Ti-S, Tokyo, Japan) using the sedimentation chamber method of Utermöhl^53^. In most cases 50 ml of the samples was settled, in some cases 10 ml. 20, 40 and 60X magnification was used and the number of view fields counted varied to obtain a minimum of 50 cells of the dominating species, i.e., with a maximum count error of ±28% according to Edler and Elbrächter^54^. Carbon biomass was determined by calculating volume from cell size^55^, which was converted to carbon using published conversion factors^56^. With this method and maximal magnification of 600X we detected mainly protists with cell diameter >2 µm.

Samples for Chl *a* were collected on 25 mm diameter GF/F filters (Whatman, GE Healthcare, Little Chalfont, UK). The volume filtered was noted. Chl *a* was extracted in 100% methanol for 12 h at 5 °C in the dark and subsequently measured using a Turner Fluorometer 10-AU (Turner Design, Inc.). Phaeopigments were measured by acidifying the sample with 5% HCl before measuring the fluorescence^57^. Samples to measure algal pigment composition were collected by filtering 10–1000 mL of sample through 25 mm GF/F filters, which were snap frozen in liquid nitrogen and then kept frozen at −80 °C until analysis. After an extraction step the pigments were measured using a Waters photodiode array detector (2996), Waters fluorescence detector (2475), and the EMPOWER software. The pigments were separated by reverse-phase high-performance liquid chromatography (HPLC) in a VARIAN Microsorb-MV3 C8 column (4.6 × 100 mm) using HPLC-grade solvents (Merck). For further details see Tran *et al*.^58^. Chl *a* was measured by both Fluorometer and HPLC for most samples in this study. A linear regression of all data from YI cores (n = 87) gave the relationship Chl *a*_HPLC_ = 0.65 Chl *a*_Fluorometer_ + 0.11, R^2^ = 0.83. All our reported Chl *a* concentrations were measured by fluorometer, whereas all alloxanthin: Chl *a* ratios were calculated from alloxanthin and Chl *a* measured by HPLC.

### Photosyntetic response measured by fluorescence kinetics

The physiological status and light response of the photosynthetic apparatus of *M. rubrum* in samples from the refrozen lead ice-water interface were assessed using *in vivo* Chl *a* fluorescence kinetics measured with a Pulse Amplitude Modulation (PAM) fluorometer (Phyto-PAM, Walz, Germany). Samples were kept in a fridge with temperature in the range 1–2 °C and dark-acclimated for 30 min prior to measurement. The maximum quantum yield of fluorescence of photosystem II (Φ_PSIImax_) was measured with the saturation pulse method^31^. Rapid Light Curves (RLC) in which the quantum yield of fluorescence in the light (Φ_PSII_) was measured by illuminating the sample with actinic light increasing stepwise from 1 to 900 µmol photons m^−2^ s^−1^ in 13 steps at 20-second intervals, were used to assess the light response of the algae. The first measurement was after dark-acclimation, i.e. Φ_PSIImax_. Relative electron transfer rate (rETR) was calculated by Φ_PSII_ × E, where E is the actinic irradiance. The photosynthesis-light function of Webb *et al*.^59^ was fitted to the rETR data as a function of the incident actinic light:

1 $${\rm{rETR}}\,={{\rm{rETR}}}_{{\rm{\max }}}[1-{{\rm{e}}}^{(-\frac{{\rm{\alpha }}E}{{{\rm{rETR}}}_{{\rm{\max }}}})}]$$

where α is the initial slope of the curve, i.e. the photosynthetic efficiency, and rETR_max_ is the curve asymptote, i.e. the maximal rETR. We did not observe inhibition of rETR at high irradiance so we did not add an inhibition term to the equation.

### Ice-ocean boundary layer dynamics

The ice-ocean boundary layer may be thought of as three different vertical zones^8^: (1) a laminar, molecular sub-layer (~0–1 mm thick) close to the sea ice-ocean interface, where the velocity varies linearly with depth; (2) a logarithmic turbulent layer (~1–3 m thick) below the laminar sub-layer, with constant stress and where the velocity varies logarithmically with depth; (3) a turbulent, thicker outer layer (~10 m thick), where the velocity is affected by the Coriolis effect. In this study we focus on the first two layers closest to the ice-water interface: the laminar sub-layer and the logarithmic turbulent layer. The surface shear stress in the ice-water interface^60^ is defined as:

2 $$\tau =\rho {u}_{\ast }^{2}$$

where ρ is the density of the water and u_*_ is the frictional or shear velocity at the boundary layer, which provides a scale for turbulence strength and for the laminar boundary layer thickness. The surface stress *τ* in the vicinity of the sea ice, which is dominated by viscous (as opposed to inertial) forces, may also be given by White^61^:

3 $$\tau =\mu \frac{du}{dz}$$

where *µ* is the dynamic viscosity of seawater, which at 0 °C is *µ* = 1.8 × 10^–2^ g cm^−1^ s^−1^. Additionally, the magnitude of the surface stress is related to the drag force of the geostrophic fluid under a boundary (e.g., further from the sea ice) as:

4 $$\tau =\rho {C}_{d}{u}_{g}^{2}$$

where *C*_*d*_ is the dimensionless geostrophic drag coefficient, taken here as 5.5 × 10^−3^ and *u*_*g*_ is the geostrophic flow away from the boundary, also referred to as the free stream velocity relative to the sea ice velocity. From (2) and (4), the frictional velocity may be estimated as:

5 $${u}_{\ast }=\sqrt{{C}_{d}}\,{u}_{g}$$

with the thickness of the laminar sub-layer being^8^:

6 $${\delta }_{lsl}=\frac{\vartheta }{k\,{u}_{\ast }}$$

where $\vartheta$ is the kinematic viscosity coefficient equal to the dynamic viscosity, $\mu$, divided by the density of seawater, $\rho$, taken here as $\rho$ = 1028 kg m^−3^, yielding $\vartheta$ = $\mu /\rho$ = 1.78 × 10^−2^ cm^2^ s^−1^; and *k* is the dimensionless Von Kármán’s constant, equal to 0.41. Following Eq. 3, the velocity structure within the laminar sub-layer (from *z* = 0 to *z* = *δ*_*lsl*_) varies with depth as:

7 $$u(z)=\frac{{u}_{\ast }^{2}z}{\vartheta }\,{\rm{for}}\,z\le {\delta }_{lsl}$$

Right below the laminar sub-layer, in the logarithmic layer (i.e., from *z* = *δ*_*lsl*_ to z = *δ*_*sl*_ ~ 1–3 m), turbulent forces become more important than viscous forces. This log layer follows the “law of the wall”, where the velocity varies logarithmically with depth (down to typically about 2–3 m below the sea ice^8^, following:

8 $$u(z)=\frac{{u}_{\ast }}{k}\,\mathrm{ln}\,\frac{z}{{z}_{o}}\,{\rm{for}}\,{\delta }_{lsl}\ge z\le {\delta }_{sl}$$

where *z*_*o*_ is a surface length scale related to the roughness elements of the surface in the ice-ocean boundary. Previous findings from laboratory experiments^62^ suggest that ${z}_{o}=\frac{{h}_{s}}{30}$, where *h*_*s*_ is the characteristic height of roughness elements, whereas ${z}_{o}=0.11\frac{\vartheta }{{u}_{\ast }}$ for a very smooth sea ice surface. The free-stream velocity *U*_*∞*_ measured at 20 m, relative to the sea ice, was on average 11 cm s^−1^, often weaker, and with a few events reaching up to approximately 30 cm s^−1^ (Fig. S1). We start with the simplest scenario of a smooth sea ice bottom, and assume that no sea-ice melt/growth was occurring when these samples were taken. We select 4 observed values of *U*_*∞*_: (a) 5 cm s^−1^; (b) 10 cm s^−1^ (representative of the mean value 11 cm s^−1^); (c) 20 cm s^−1^ and (d) 30 cm s^−1^. Using Eqs 6 and 7, we solve for the frictional velocity u_*_ and the thickness of the laminar sub-layer *δ*_*lsl*_. We then solve for the velocity profiles both at the laminar sub-layer (linear) and at the logarithmic layer.

## Supplementary information

Supplementary material for: A red tide in the pack ice of the Arctic Ocean

## Data Availability

The data sets used in this study are publicly available from the Norwegian Polar Data Centre (https://data.npolar.no): N-ICE 2015 surface and under-ice spectral shortwave radiation data [Taskjelle *et al*.]^63^; N-ICE 2015 water column biogeochemistry [Assmy *et al*.]^64^; N-ICE 2015 ocean microstructure profiles [Meyer *et al*.]^65^; N-ICE 2015 phytoplankton and ice algae taxonomy and abundance [Olsen *et al*.]^66^; N-ICE 2015 total snow and ice thickness data from EM31 [Rösel *et al*.]^67^.
