# Supplementary material for: A red tide in the pack ice of the Arctic Ocean
Source: Sci Rep. 2019 Jul 2;9:9536. doi: 10.1038/s41598-019-45935-0 (PMC6606610; doi:10.1038/s41598-019-45935-0)
Supplement: Supplementary file 1 — Supplementary material for: A red tide in the pack ice of the Arctic Ocean [file 41598_2019_45935_MOESM1_ESM.pdf]

Supplementary material for

A red tide in the pack ice of the Arctic Ocean

Lasse M. Olsen[1, 2*], Pedro Duarte[1], Cecilia Peralta-Ferriz[3], Hanna M. Kauko[1], Malin Johansson[4], Ilka Peeken[5], Magdalena Różańska-Pluta[6], Agnieszka Tatarek[6], Jozef Wiktor[6], Mar Fernández-Méndez[1, 7], Penelope M. Wagner[8], Alexey K. Pavlov[1, 6, 9], Haakon Hop[1, 10], Philipp Assmy[1]

[1] Norwegian Polar Institute, Fram Centre, Tromsø, Norway

[2]Department of Biological Sciences, University of Bergen, Bergen, Norway

[3] Polar Science Center, Applied Physics Laboratory, University of Washington, Seattle, WA, USA

[4] Department of Physics and Technology, University of Tromsø - The Arctic University of Norway, Tromsø, Norway

[5] Alfred Wegener Institute Helmholtz Center for Polar and Marine Research, Bremerhaven, Germany

[6] Institute of Oceanology, Polish Academy of Sciences, Sopot, Poland

[7]Biological Oceanography, GEOMAR Helmholtz Centre of Ocean Research Kiel, Kiel, Germany

[8] Norwegian Ice Service, Norwegian Meteorological Institute, Tromsø, Norway

[9] Akvaplan-niva, Fram Centre, Tromsø, Norway

[10] Department of Arctic and Marine Biology, Faculty of Biosciences, Fisheries and Economics, University of Tromsø - The Arctic University of Norway, Tromsø, Norway

[*]corresponding author: lasse.mork.olsen@npolar.no

Supplementary Section 1: Supplementary Figures S1.1 to S1.4

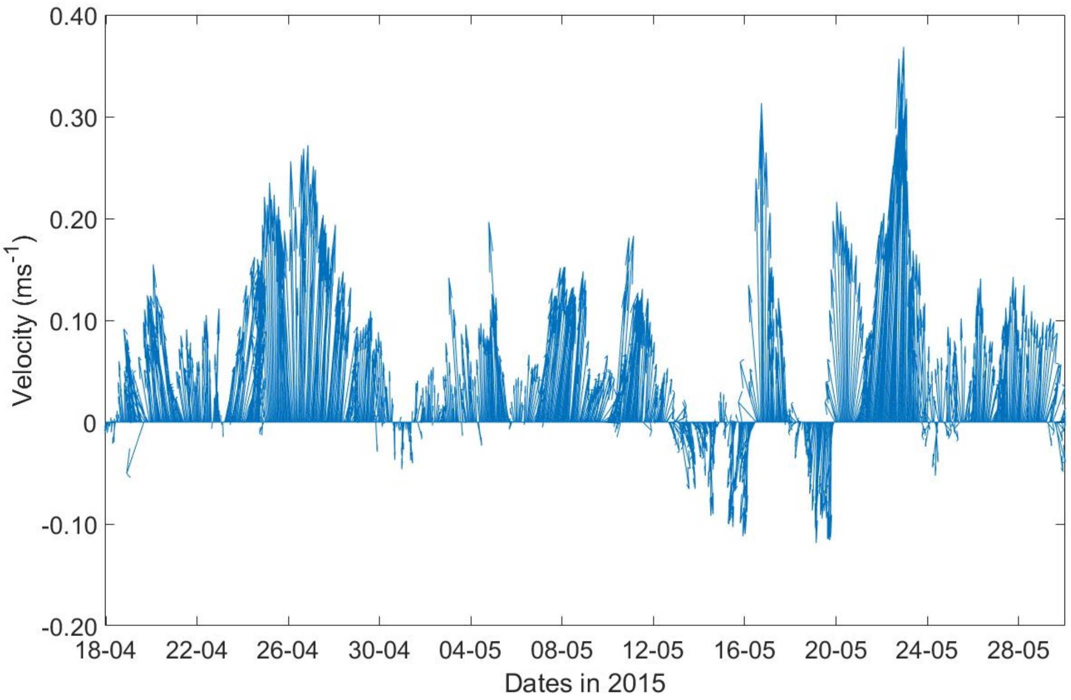

Figure S1.1: The relative velocity and direction of the water relative to the ice measured by ADCP profiler centered at 23 m depth between 18 April and 30 May. North is upwards. The apparent northward direction of the water is mainly due to the faster wind-driven southward movement of the ice.

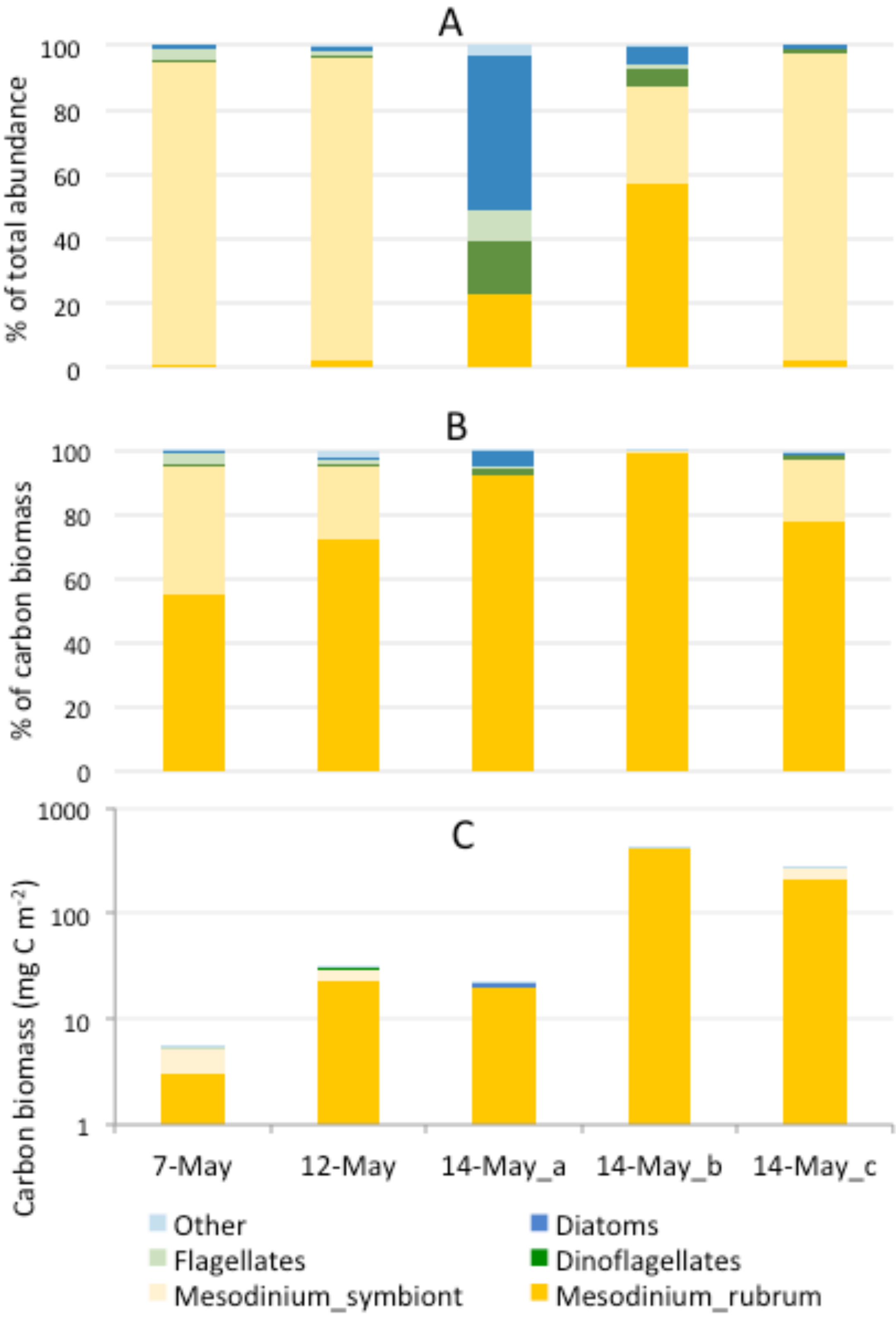

Figure S1.2: Relative abundance A) and carbon biomass B), and absolute carbon biomass C) of *M. rubrum* and other protist groups in slurp samples from the ice-water interface of the young ice of the refrozen lead. 3 replicates for 14 May. See supplementary section 4 for species list.

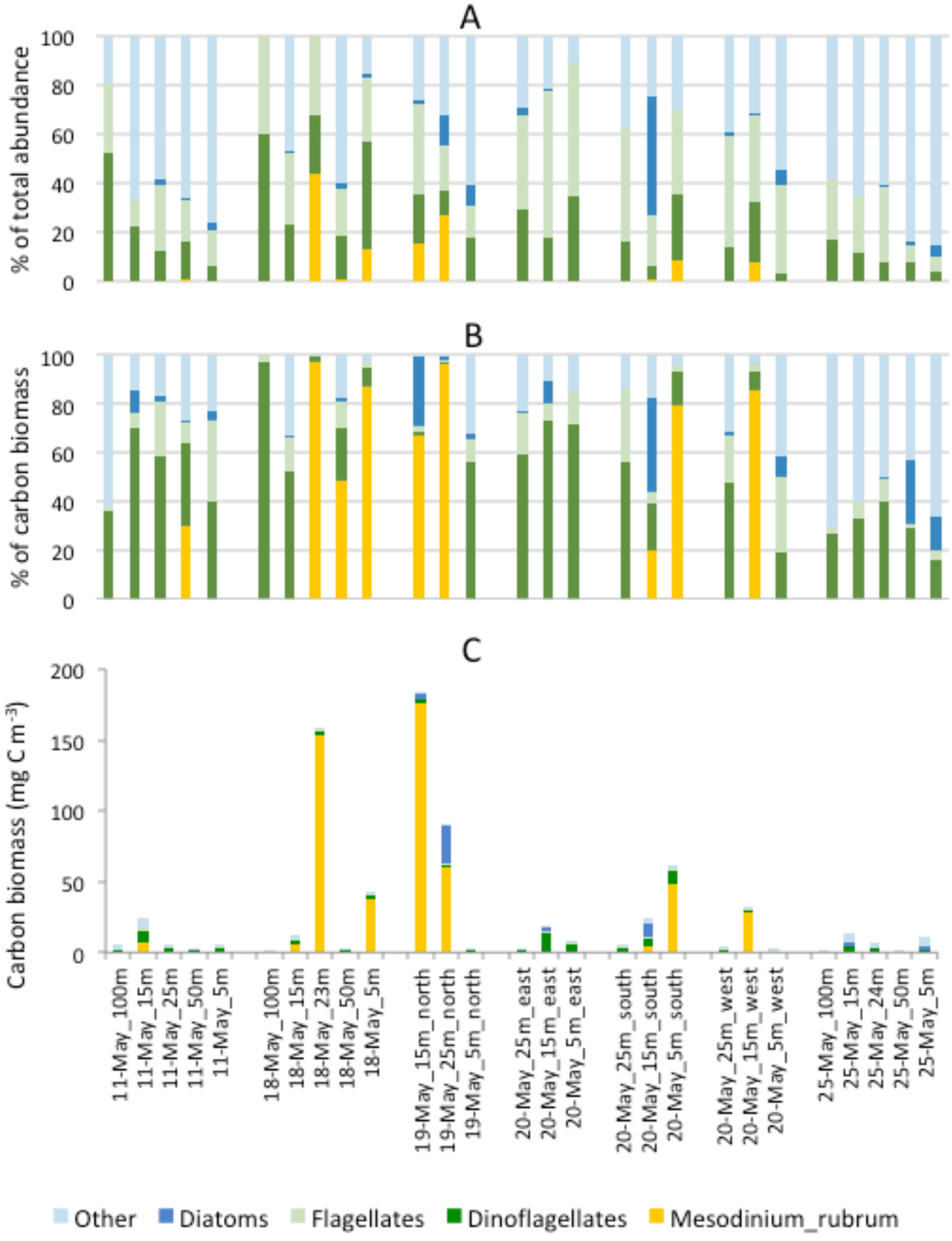

Figure S1.3: Relative abundance A) and carbon biomass B), and absolute carbon biomass C), of *M. rubrum* and other protist groups in depth profile samples from the water column under Floe 3 on 11, 18 and 25 May, and at the spatial sampling points on 19 and 20 May. See supplementary section 4 for species list.

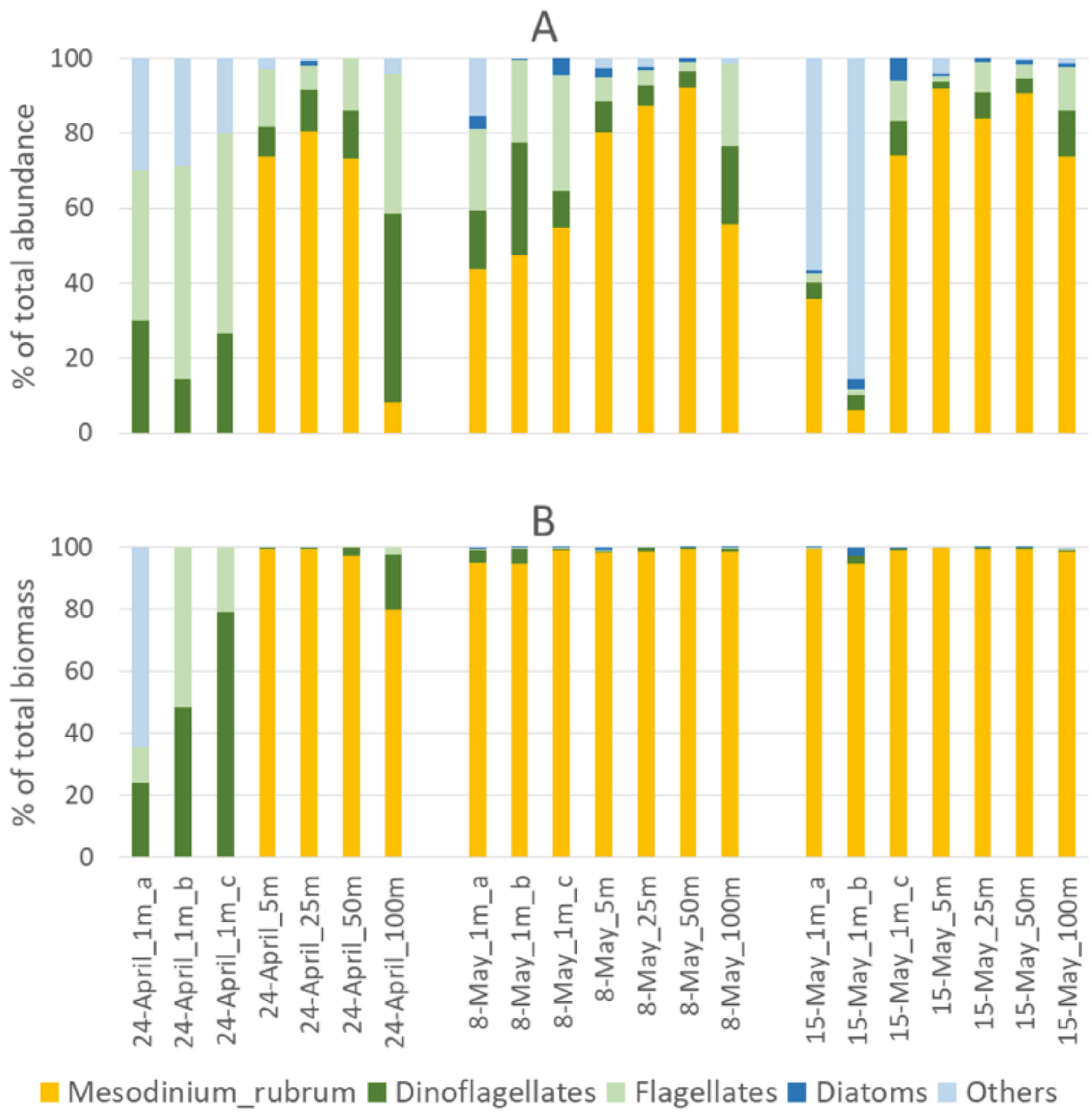

Figure S1.4: Relative abundance A) and carbon biomass B) of *M. rubrum* and other protist groups in sediment trap samples. Species list in supplementary section 4.

Supplementary section 2: Ice type classification from Advanced Land Observing Satellite 2 synthetic aperture radar scene

Method

Sea ice surface types defined as; open water, new and young ice (YI), smooth and deformed first-year ice (FYI) / multi-year ice (MYI) were evaluated using a synthetic aperture radar (SAR) ALOS-2 Palsar-2 scene from May 18 at 20:25 UTC (see Figure S2.1). The satellite scene is an L-band fully polarimetric image with a spatial resolution of approximately 5 m and an aerial extent of 40 km x 70 km. L-band SAR images have been used by e.g., Nakamura et al 2005, Casey et al 2016, Johansson et al 2017 to classify sea ice types.

The satellite scene used here was first calibrated using the metadata information provided in Shimada et al (2015) and then segmented using the "extended polarimetric feature space" algorithm (Doulgeris and Eltoft, 2010; Doulgeris, 2013). The segmentation uses statistical properties of texture features and separates them into distinct classes based on polarimetric and textural properties for each pixel. The segments were assigned labels to represent a specific sea ice feature by researchers at the Norwegian Ice Service following procedures used and documented in MANICE (Canadian Ice Service (CIS) Meteorological Service of Canada, Environment Canada, 2005). The scheme has demonstrated the capability to discriminate between thin ice up to approximately 0.5 m and smooth and deformed ice (Johansson et al., 2017 and Assmy et al. 2017). The classified sea ice type areas were further evaluated using sea ice and snow thickness measurements combined with photographs measured from a helicopter on May 18 between 11:41 and 12:22 UTC (King et al, 2016).

Results

In the Arctic, backscatter values can be used as an indication of the type of sea ice based on surface roughness and volume scattering in the ice. Higher backscattering values (brighter surface features) suggest older and thicker ice, whereas lower values (darker surface features) are characteristic of new and young ice and open water (Oliver and Quegan, 2004). During the melt season surface flooding on the sea ice influences the signal return where older and younger ice types can have the same return signal so it is necessary to consider visible characteristics in surface features.

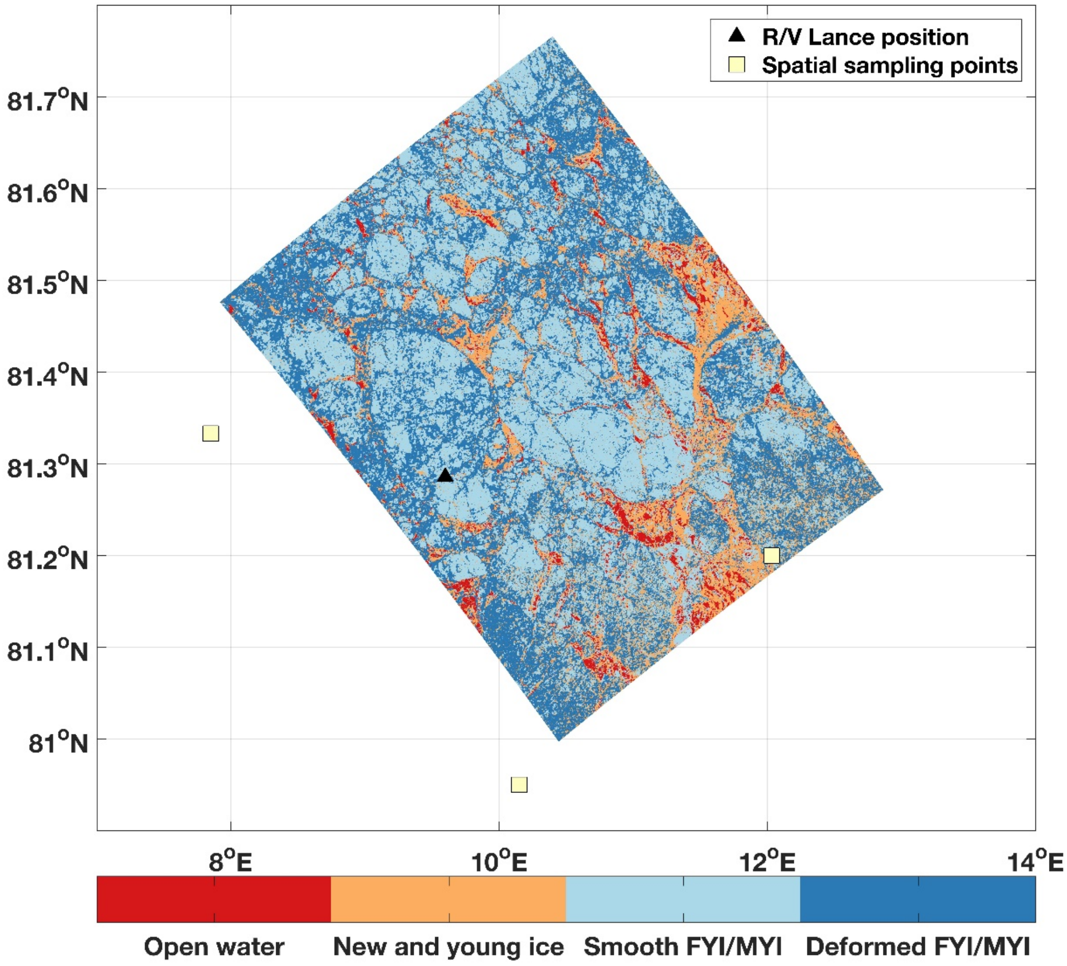

Figure S2.1: Ice classified on ALOS-2 SAR image from 18 May; new and young ice made up 10.2% of the total satellite scene areal extent, open water was 5.4% and the smooth FYI/MYI constituted 42.3% and the rough FYI/MYI was 42.1%. The black triangle shows the position of R/V Lance, and the yellow squares show the positions for regional sampling by helicopter on 20 May, see Fig. 1 for details.

Well-defined surface features from the SAR image allowed ice types to be easily discernible when applying the segmentation. Smooth FYI/MYI were represented by large polygonal shapes normally formed as the pack ice begins to break up and converge/diverge with other ice floes during the melt season. Deformed sea ice structures are clear throughout the image between and within the floes, as a result of this dynamic period during the melt season. There is a distinct separation between the FYI/MYI ice and young ice/open water areas with minimal mixing between the two types.

Based on the sea ice type classification the percentage of open water was 5.4%, new and young ice made up 10.2% of the total satellite scene areal extent, the smooth FYI/MYI constituted 42.3% and the rough FYI/MYI was 42.1% (Fig. S2.1).

References

Assmy, P., M. Fernández-Méndez, P. Duarte, A. Meyer, A. Randelhoff, C.J. Mundy, L.M. Olsen, H. Kauko, A. Bailey, M. Chierici, L. Cohen, A.P. Doulgeris, J.K. Ehn, A. Fransson, S. Gerland, H. Hop, S.R. Hudson, N. Hughes, P. Itkin, G. Johnsen, J. King, B.P. Koch, Z. Koenig, S. Kwasniewski, S.R. Laney, M. Nicolaus, A. Pavlov, C.M. Polashenski, C. Provost, A. Rösel, M. Sandbu, G. Spreen, L.H. Smedsrud, A. Sundfjord, T. Taskjelle, A. Tatarek, J. Wiktor, P.M. Wagner, A. Wold, H. Steen and M.A. Granskog (2017): Leads in Arctic pack ice enable early phytoplankton blooms below snow-covered sea ice. Scientific Reports 7, No. 40850, doi: 10.1038/srep40850

Canadian Ice Service (CIS) Meteorological Service of Canada Environment Canada (2005). Manual of Standard Procedures for Observing and Reporting Ice Condition (MANICE), Revised Ninth Edition. ISBN 0-660-62858-9:146.

Casey, J.A., Howell, S.E., Tivy, A., and Haas, C. (2016) Separability of sea ice types from wide swath C- and L-band synthetic aperture radar imagery acquired during the melt season. Remote Sensing of Environment, 174, 314–328, doi: 10.1016/j.rse.2015.12.02

Doulgeris, A.P. (2013). A Simple and Extendable Segmentation Method for Multi-Polarisation SAR Images. in POLinSAR 2013 (Frascati, Italy), 8 pp.

Doulgeris, A.P., and Eltoft, T. (2010). Scale mixture of Gaussian modelling of polarimetric SAR data. EURASIP J. Appl. Signal Proc., 12. doi:10.1155/2010/874592.

Johansson, A.M., King, J.A., Doulgeris, A.P., Gerland, S., Singha, S., Spreen, G., Busche, T., 2017. Combined observations of Arctic sea ice with near-co-incident co-located X-band, C-band, and L-band SAR satellite remote sensing and helicopter-borne measurements. J. Geophys. Res. Oceans 122, 669–691. Doi: 10.1002/2016JC012273.

King, J.A., Gerland, S., Spreen, G., and Bratrein, M. (2016), N-ICE2015 sea-ice thickness measurements from helicopter-borne electromagnetic induction sounding [Data set]. Norwegian Polar Institute, doi: 10.21334/npolar.2016.aa3a5232.

Nakamura, K., Wakabayashi, H., Naoki, K., Nishio, F., Moriyama, T., and Uratsuka, S., 2005. Observation of sea-ice thickness in the Sea of Okhotsk by using dual-frequency and fully polarimetric airborne SAR (Pi-SAR) data. IEEE Trans. Geosci. Remote Sens. 43 (11), 2460–2469. Doi: 10.1109/TGRS.2005.853928.

Oliver, C and Quegan, S. (2004). Understanding Synthetic Aperture Radar Images. SciTech Publishing Inc., Raleigh, USA.

Shimada M, M W, Motooka T, Kankaku Y and Suzuki S (2015) Calibration and validation of the PALSAR-2. IEEE International Geoscience and Remote Sensing Symposium, IGARSS 2015, Milan, Italy, 26–31 July

Supplementary Section 3: Effects of roughness on the ice-ocean boundary layer dynamics

When the roughness elements (measured by roughness thickness, h_s) (Equation 7) are smaller than the laminar sub-layer thickness, δ_{lsl}, then the logarithmic layer does not feel this roughness, and the flow in this layer is denoted smooth turbulent, or hydrodynamically smooth. Alternatively, when the roughness thickness, h_s, exceeds the thickness of the laminar sub-layer, δ_{lsl}, the roughness is felt by the flow in the logarithmic layer, and this flow then becomes rough turbulent, or hydrodynamically rough. Transition between the two types of regimes may also occur. The smooth turbulent regime occurs when the roughness Reynolds number $R^* \leq 5$, where $R^* = \frac{h_s u_*}{\vartheta}$, whereas the fully rough turbulent regime occurs when $70 \leq R^* \leq 100$. The turbulent flow in the surface layer is in transition when $5 < R^* < 70$ (Nikuradse 1933). Figure S3.2 illustrates these regimes estimated for each of the free-stream velocities of U_∞=5 cm s^{-1} and U_∞=10 cm s^{-1}.

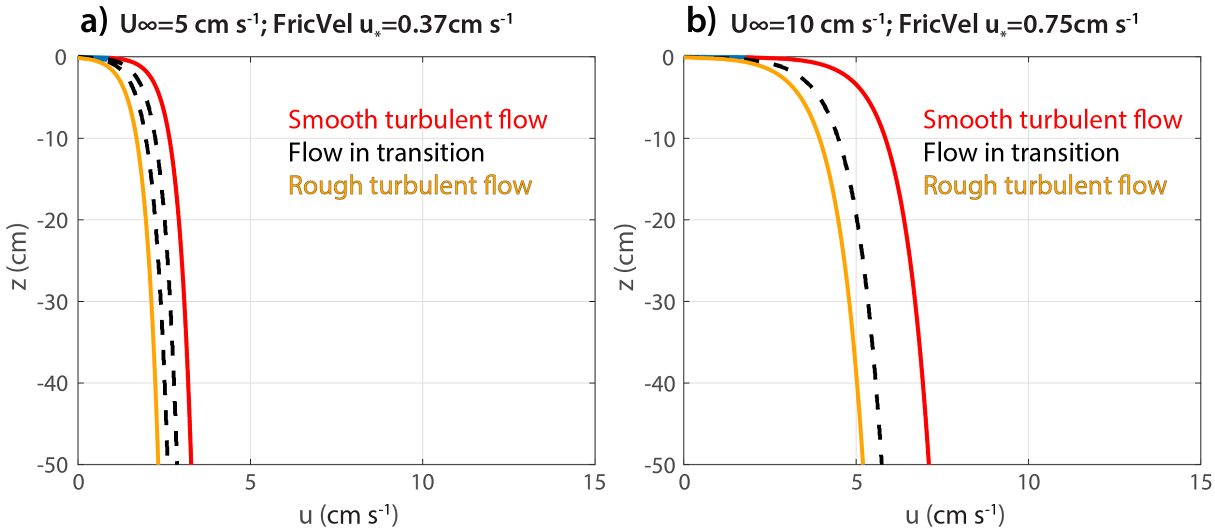

Figure S3.1: Velocity profiles in the logarithmic layer below rough ice, considering stream velocities of a) U_∞=5 cm s^{-1} and b) U_∞=10 cm s^{-1}. a) Turbulent flow for (red) roughness value h_s≤0.1 cm, which is smaller than laminar sub-layer thickness δ_{lsl}=0.12 cm, hence denoted smooth turbulent flow (roughness Reynolds number $R^* = 2$; for (yellow) roughness h_s=4 cm, which yields $R^* = 83$, and since 70<R*<100, it is denoted rough turbulent flow; and for (dash black) roughness values of h_s=2 cm and h_s=3 cm, which yield R*=42 and R*=62, respectively, hence the flow is in transition from smooth to rough turbulent. b) Similar to a), but for (red) values of h_s ≤0.05 cm (smaller than δ_{lsl}=0.06 cm, R^*=2); for (yellow) h_s=2 cm (R^*=83); for (dash-black) h_s=1 cm, which yields R^*=42.

When $h_s > \delta_{lsl}$ (i.e., in both transitional and rough turbulent flows), the laminar sub-layer is either fully destroyed or, according to Bijker (1971), simply becomes much thinner than that in the smooth sea ice case. Therefore roughness elements would presumably reduce the possibility for the organisms to be sheltered in the even thinner (if still existent) laminar sub-layer. However, Figure S3.2 also shows that as the roughness elements increase in size, the flow in the surface (logarithmic) layer tends to slow down, as drag forces act on the turbulent flow (Equation 7). One could then speculate that the slower flow may counteract the thinning of the sub-layer in some regions, potentially leading to calm regions where *M. rubrum* cells could enter a still-existing laminar sub-layer and be retained closer to the ice-water interface from the water column below. We do not think this is the case either, because M. *rubrum* cells were present during sea ice growth (i.e., period in which the thin laminar sub-layer is disrupted) and absent after sea ice growth had stopped (i.e., period in which the thin laminar sub-layer is able to form).

References:

Bijker, E. W. (1971), Longshore transport computation, Journal of Waterways, Harbours and Coastal Engineering Division. ASCE, Volume 97, WW 4. pp687-701.

Nikuradse, J. (1933). Laws of flow in rough pipes. Tech Rep. NACA Technical Memorandum 1292. National Advisory Commission for Aeronautics. Washington, DC, USA.

Supplementary Section 4: Grouped species list

	Young ice	Water column	Sediment traps	Slurp samples
Mesodinium rubrum	X	X	X	X
Mesodinium rubrum free chloroplast	X			X
**DINOFLAGELLATES**				
Alexandrium sp.	X	X	X	
Amphidinium crassum	X	X		
Amphidinium sp.		X		
Azadinium sp.	X			X
Azadinium spinosum		X	X	
Cochlodinium sp.	X	X		
Dicroerisma psilonereiella			X	
Dinoflagellate cysts	X			
Dinophyceae indet. 40-50um			X	
Dinophyceae indet. cyst				X
Dinophyceae indet. encysting		X	X	
Dinophycean indet. 10-20um	X			
Dinophysis contracta			X	
Gymnodinium arcticum	X	X	X	
Gymnodinium galeatum	X	X	X	X
Gymnodinium gracilentum	X	X	X	
Gymnodinium gracilentum				X
Gymnodinium simplex	X	X	X	
Gymnodinium sp. <10um	X			X
Gymnodinium sp. 10-20um	X	X	X	X
Gymnodinium sp. 20-30um	X	X		
Gymnodinium sp. 30-40um	X			
Gymnodinium sp. 7-10um		X	X	
Gymnodinium sp. cf. G. wulffii		X	X	
Gymnodinium sp. cf. G.arcticum				X
Gymnodinium sp. cyst	X			
Gymnodinium wulffii				X
Gymnodinium wulfii	X			
Gymnodinium/Gyrodinium 10-20um	X			
Gyrodinium estuariale			X	
Gyrodinium flagellare	X	X		
Gyrodinium fusiforme	X			
Gyrodinium sp	X			
Gyrodinium sp.		X		
Gyrodinium sp. 20-30um		X		
Heterocapsa sp.	X	X	X	X
Heterocapsa sp./*Polarella glacialis*	X			
Heterocapsa arctica	X	X	X	X
Heterocapsa rotundata	X	X		X
Heterocapsa sp. cf. H. *niei*	X	X	X	
Lessardia elongata	X	X	X	X

Oxyrrhis sp.	X	X		
Oxytoxum gracile		X		
Polarella glacialis	X			X
Polarella glacialis cyst	X		X	
Prorocentrum minimum	X	X	X	X
Protoperidinium brevipes		X	X	X
Protoperidinium monacanthum			X	
Protoperidinium pellucidum		X		
Protoperidinium sp.	X		X	
Thecate dinophyceae indet.			X	
Thecate dinophyceae indet. 10-20um	X			X

FLAGELLATES

Anisonema sp.	X			
Bicosta minor	X			
Bicosta spinifera	X			
Bifagellatae ident. 7-10um	X			
Bifagellatae indet. 3-7um	X			
Biflagellatae indet. >10um	X			
Biflagellatae indet. 3-7um				X
Biflagellates indet. 3-7um		X	X	
Biflagellates non det. ~3um		X		
Chlorophyceae indet. >10um	X			
Chlorophyceae indet. 3-7um	X			
Chlorophyceae indet. 7-10um	X			X
Choanoflagellatea indet.	X			
Chrysophyceae cyst	X			
Cryptomonas sp.	X			X
Cryptophyceae indet.	X	X	X	
Cryptophyceae indet. 11-15um	X			
Cryptophyceae indet. 3-7um	X			
Cryptophyceae indet. 7-10um	X			X
Dictyocha speculum		X		
Dinobryon balticum			X	
Dinobryon faculiferum	X	X		
Dinobryon sp.	X	X		
Dinobryon sp. statocysts	X			
Dinobryon sp. statospores		X	X	
Euglenoidea indet.	X	X	X	
Eutreptiella sp.	X			
Flagellates indet.	X			
Flagellates indet. <5um				X
Flagellates indet. >10um	X			
Flagellates indet. ~3um		X	X	
Flagellates indet. 3-7um	X	X	X	X
Flagellates indet. 7-10um	X	X		X
Heterotrophic flagellate	X			
Leucocryptos marina	X	X	X	
Leucocryptos remigera	X			
Mantoniella sp.	X			

Monosiga marina	X			
Nephroselmis sp.		X		
Pavlova lutheri	X			
Plagioselmis sp.		X	X	
Prasinophyceae indet 6-10um		X	X	
Prasinophyceae indet. 7-10um	X			
Pyramimonas nansenii	X			
Pyramimonas sp.	X	X		X
Pyramimonas sp. cf. P. *nansenii*		X	X	X
Pyramimonas sp. cf. P. *virginica*	X		X	X
Rhodomonas sp.	X			
Teleaulax acuta	X	X		
Teleaulax amphioxeia		X		
Teleaulax sp.	X	X	X	
Telonema subtile	X	X	X	
Unifagellatae ident. 3-7um	X			
Unifagellatae ident. 7-10um	X			
Unifagellates indet. 3-7um		X		
Uniflagellatae indet. 11-15um	X			

DIATOMS

Actinocyclus sp.			X	
aff *Catenula* sp.	X			
Asteromphalus hyalinus			X	
Attheya septentrionalis	X	X		X
Bacterosira bathyomphala	X			
Centriceae indet.			X	
Centriceae indet. <30um	X	X		X
Centriceae indet. >30um	X	X		
Ceratoneis closterium	X	X	X	X
Chaetoceros furcellatus	X			
Chaetoceros simplex	X			
Chaetoceros socialis	X			X
Chaetoceros sp.	X		X	
Chaetoceros tenuissimus	X	X		X
Conticribra weissflogii	X			
Entomoneis kjellmanii	X		X	
Entomoneis paludosa	X			X
Entomoneis sp.		X		
Fossula arctica	X			X
Fragilariopsis cylindrus	X	X	X	X
Fragilariopsis oceanica	X	X		
Fragilariopsis reginae-jahniae	X			
Fragilariopsis sp.				X
Fragilariopsis sp. cf. *F.nana*	X			
Hantzschia weyprechtii	X	X		
Haslea crucigeroides	X			
Haslea sp.	X			
Haslea sp. cf. *H.spicula*	X			
Navicula algida	X			

Navicula directa	X			X
Navicula distans	X	X		
Navicula kariana	X			
Navicula pelagica	X	X		X
Navicula sp. <30um	X			
Navicula sp. >30um	X	X	X	
Navicula sp. cf. *N. gelida*	X			
Navicula superba	X			
Navicula transitans	X	X		X
Navicula trigocephala	X			
Navicula vanhoeffenii	X			
Nitzschia arctica	X			
Nitzschia frigida/neo-frigida	X	X	X	X
Nitzschia leavissima	X	X		
Nitzschia longissima	X		X	
Nitzschia promare	X			
Nitzschia sp.		X	X	
Nitzschia sp. <20um	X			
Nitzschia sp. >30um	X			
Pauliella taeniata	X			
Pennales indet. <10um	X			
Pennales indet. <30um		X	X	X
Pennales indet. >30um	X		X	X
Pennales indet. 10 -30um	X			
Pinnularia semiinflata	X			
Porosira glacialis	X			
Pseudo-nitzschia cf. *P. granii*				X
Pseudo-nitzschia delicatissima/pseudodelicatissima	X	X	X	X
Pseudo-nitzschia granii	X	X		X
Pseudo-nitzschia seriata	X			
Pseudo-nitzschia sp.	X	X		X
Pseudogomphonema arcticum	X			
Rhizosolenia hebetata			X	
Stauroneis radissonii	X			
Stenoneis obtuserostrata	X			
Synedropsis hyperborea	X		X	
Thalassiosira bioculata	X	X	X	X
Thalassiosira gravida/antarctica	X	X		
Thalassiosira hyalina	X			X
Thalassiosira nordenskioeldii	X			
Thalassiosira sp.	X		X	X

OTHER

Algirosphaera robusta	X	X	X	
Ciliophora indet.		X	X	
Ciliophora indet.	X			X
Ciliophora indet. 10-20um	X	X	X	
Ciliophora indet. 20-30um	X	X		X
Ciliophora indet. 30-40um	X			
Ciliophora indet. 40-50um	X			

Ciliophora indet. 70-80um	X			
Ciliophora indet. 80-90um	X			
Ciliophora indet. 90-100um	X			
Ciliophora indet. cyst	X			
Coccolithales indet.	X			
Coccolithales indet. >15um	X			
Coccolithales indet. 11-15um	X			
Coccolithales indet. 3-7um	X			
Coccolithales indet. 6-10um		X	X	X
Coccolithales indet. 7-10um	X			X
Cyst	X			
Cyst indet.			X	
Emiliania huxleyi	X	X		
Euplotes sp.	X			
Incertains cyst			X	
Incertains taxa			X	
Leegaardiella sol	X			X
Lohmanniella oviformis	X	X		
Phaeocystis pouchetii	X	X	X	X
Radiozoa indet.			X	
Salpingella secata			X	
Strombidium sp.	X	X		
Uronema marinum		X		
